# Four-Level Micro-Via Technology (4LµV) for ASIC Integration in Active Flexible Sensor Arrays

**DOI:** 10.3390/s22134723

**Published:** 2022-06-22

**Authors:** Maolei Zhou, Chresten von der Heide, Andreas Dietzel

**Affiliations:** Institute of Microtechnology, Technische Universität Braunschweig, 38124 Braunschweig, Germany; c.von-der-heide@tu-braunschweig.de (C.v.d.H.); a.dietzel@tu-braunschweig.de (A.D.)

**Keywords:** micro-via, copper electroplating, flexible sensor arrays, chip-in-foil-integration

## Abstract

Systems-in-foil with multi-sensor arrays require extensive wiring with large numbers of data lines. This prevents scalability of the arrays and thus limits the applications. To enable multiplexing and thus reducing the external connections down to few digital data links and a power supply, active circuits in the form of ASICs must be integrated into the foils. However, this requires reliable multilayer wiring of the sensors and contacts for chip integration. As an elegant solution to this, a new manufacturing process for multilayer wiring in polyimide-based sensor foils has been developed that also allows ASIC chips to be soldered. The electrical four-level micro-via connections and the contact pads are generated by galvanic copper deposition after all other process steps, including stacking and curing of polyimide layers, are completed. Compared to layer by layer via technology, the processing time is considerably reduced. Because copper plating of vias and solderable copper contact pads happens as the final step, the risk of copper oxidation during polyimide curing is completely eliminated. The entire fabrication process is demonstrated for six strain sensor nodes connected to a surface-mounted ASIC as a detecting unit for sensing spatially resolved bending states. Each sensor node is a full-bridge configuration consisting of four strain gauges distributed across interconnected layers. The sensor foil allows bending of +/−120° without damage. This technology can be used in future for all kinds of complex flexible systems-in-foil, in particular for large arrays of sensors.

## 1. Introduction

In recent years, the design of sensory systems in the form of flexible foils has significantly evolved in many application areas, allowing tactile sensing [1,2], temperature [3] and bending measurements [4,5,6,7], as well as medical monitoring [3,5,7,8]. In the field of medical sensors, skin-attachable designs are used to receive bio signals [5,7,8,9]. Most applications require multilayer wiring, which demands vertical interconnects by micro-vias. Two-level-interconnections in flexible foils have been reported [2,7,10]. Through-silicon vias (TSVs) used in the chip technology are characterized by high aspect ratios to save chip area [11,12]. In contrast, the flexible sensor arrays have far fewer restrictions in terms of space consumption but require materials and geometries that prevent damage by repeated bending and twisting. Some active systems use chips encapsulated in film, which results in a thicker finished product and requires additional packaging for a more attractive appearance [5,8]. Integrated ultra-thin chips lead to thinner products and can allow repeated bending and twisting of the systems [13,14,15]. Interconnection techniques based on conductive pastes [16,17] and chip embedding using conductive adhesives have been developed [15,18]. However, temperature-stable materials and solderable metal pads are required to provide reliable solderable connections for chip integration. An appropriate substrate material for such flexible sensors is highly temperature stable polyimide, which can be processed as a liquid precursor to spin-on a temporary rigid substrate, allowing lithographic structuring [19].

Previously, a passive flexible sensor array with 36 sensor nodes to measure and reconstruct the surface deformation of the whole foil system was developed [6,7]. A remaining disadvantage is that this multi-sensor array requires extensive cabling with 72 signal lines. To enable multiplexing and thus reduce the external connections to only a few digital data links and a power supply, digital circuits in the form of ASICs must be integrated to form an active system-in-foil. To integrate thin ASIC chips, contact pads on the surface of the multi-sensor foil and a complex multi-level wiring with electrical micro-vias are required. However, with a layer by layer via technology, the plated copper in the vias will experience oxidation during PI curing which will cause higher resistances and instabilities in the electrical via contacts. Protecting gas atmospheres or protective layers would add complexity to the process and may require modification of process tools. Therefore, a multi-layer process for solderable vias and ASIC contact pads is needed, where processing effort is considerably reduced and the risk of copper oxidation during polyimide curing is prevented. This will be a key technology that creates the conditions for multiplexing sensor signals in the flexible foil and drastically reduces the number of external connections.

## 2. Design and Fabrication

### 2.1. Sensor Circuit

**Figure 1:**
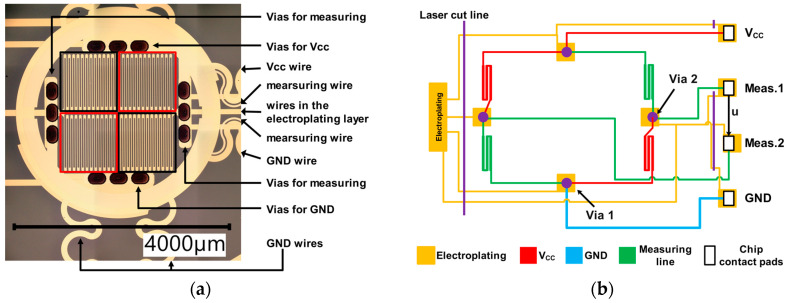
(**a**) Micrograph of a single sensor node. Strain gauges marked with the same color (black or red) are located on the same level within the multilayered device. (**b**) Schematic of the sensor nodes Wheatstone bridge circuit. Also shown are the leads for equipotential connection required during galvanization, which are afterwards cut off with a laser.

A total of 24 complex multilayer Wheatstone bridges were realized in this sensor array. Strain gauges were arranged on two levels and an electrical ground layer sandwiched between them. All electrical leads and the strain gauges were made of thin film gold for biocompatibility reasons. Each sensor node had a size of 4 mm in diameter and included four parallel aligned strain gauges. Two strain gauges in diagonally opposite arrangement and their power supply wires (V_cc_), as shown and schematically sketched in Figure 1, were on the same level within the stacked system with polyimide (PI) film interlayers. Other wires such as ground (GND) and two measuring wires were placed in a different layer. The connecting tracks exhibited a meandering shape to reduce the risk of rupture and a resistance change caused by bending the sensor film. Four electrodes (Vcc, GND, and two measuring contact pads) connected to the ASIC.

### 2.2. Multilevel Metallisation and Micro-Via Opening

Four-inch borofloat glass wafers (SCHOTT Technical Glass Solutions GmbH) were used as temporary rigid carriers during production. A 10 nm chromium adhesion layer was applied by sputter deposition (Laborsystem LS 440 S from Von Ardenne Anlagentechnik GmbH, Dresden, Germany) using a bezel to produce a 5 mm wide ring at the wafer edge providing sufficient adhesion of the PI films which were spin-coated. For soft baking the PI film, the wafers were heated at 110 °C and 155 °C for 5 min each and then cured at 350 °C in an oven (LCD1-51N-5 from Despatch Industries) for one hour in a nitrogen atmosphere before a new Au layer was sputter deposited (Laborsystem LS 440 S from Von Ardenne Anlagentechnik GmbH). The metal layers were structured using photolithography (Maskaligner EVG^®^620 from EV Group with photoresist maP 1215 from micro resist technology GmbH); the gold etching solution was made of iodine, potassium iodide and deionized water in the ratio of 1:2:20. The PI + metallization processes were repeated three times to establish the four-level architecture. The Au layers had varied thicknesses to obtain the required resistance values for each layer. Au tracks in V_cc_ and measuring lines layers were 80 nm thick to obtain a resistance value above 1.5 kΩ. In the electroplating and ground layers, the Au tracks had a thickness of 300 nm and 150 nm respectively to reduce electrical transmission losses.

**Figure 2:**
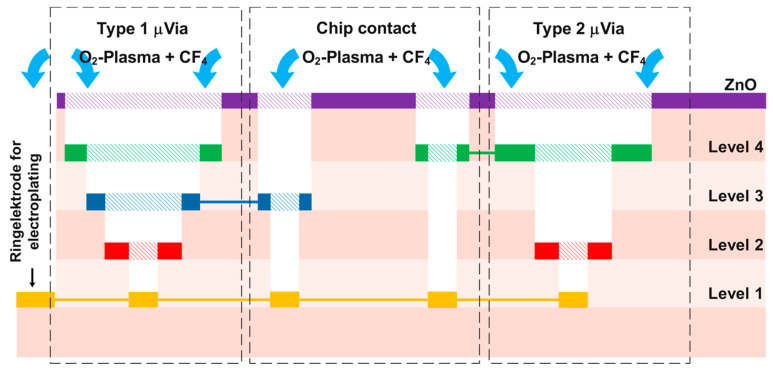
Schematic cross-sectional view of the via structure for connecting the individual layers.

**Figure 3:**
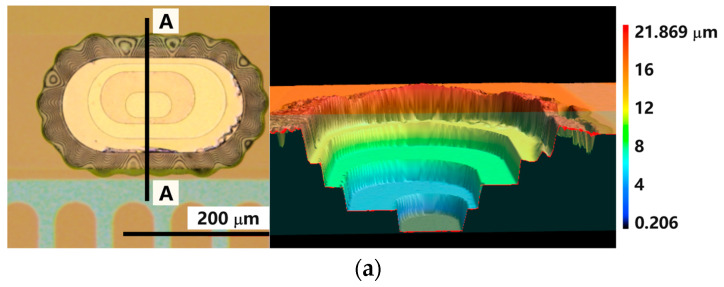

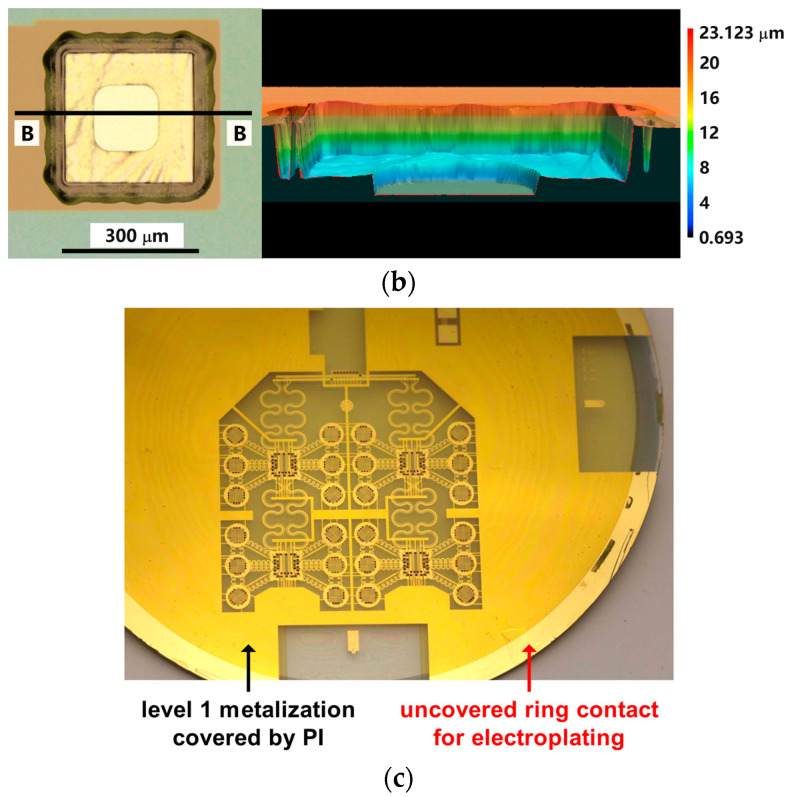
Micrographs together with 3D profiles (the digital cross-sectional cut was placed along A-A and B-B lines) as obtained by confocal laser scanning microscopy (VKX-260 from Keyence) showing a type 1 micro-via (**a**) and a chip contact hole (**b**). Fringe patterns at the edges of the ZnO hardmask indicate partial under-etching/delamination which is unproblematic for the electrical circuitry. (**c**) Picture of four entire sensor arrays each with six sensor node positions while still attached to the glass substrate. Each sensor node consists of a Wheatstone bridge with four strain gauge elements. Around the sensor array the unstructured level 1 metallization, which is covered with four levels of transparent PI coatings, becomes visible. At the outer ring, the metal is uncovered to provide the contact during electroplating.

On top of the fifth cured PI film (covering the level 4 metallization, see Figure 2) a 350 nm thick ZnO film was sputter deposited. Using photolithography (photoresist maP 1215) and 12% HCl wet etching, the ZnO was locally removed above the via positions and above the micro-via ring electrodes before dry etching in a barrel etcher (308 PC Barrel Etching System from STS Surface Technology Systems GmbH). The etch gas consisted of 80% O_2_ and 20% CF_4_. At 150 W the PI was locally removed within an hour, leaving the stepped clean Au surface and the desired hole down to the level 1 metallization as shown in Figure 3.

As a time saving alternative to the plasma etching, femtosecond laser ablation can be used to drill the micro-vias into the PI film. Since the metallization level 1 Au layer was very thin, the laser parameters had to be selected carefully to fully remove the PI of different thickness but not to damage the metal. In first tests with simplified micro-via test structures it was found that thin layers of unremoved PI were hard to avoid but with only 5 min of dry etching the gold surface could be perfectly cleaned. However, a micro-via design adaption would be required. Even with precise knowledge of the laser ablation parameters it was hard to predict the necessary process time for each via depth, which means each level needs separate tests.

### 2.3. Copper Electroplating

The wafer was fixed in an electroplating holder which couples the level 1 metallization to the current source (Source Meter Model 2450 from Keithley Instruments, LLC) via a circular cathode and then immersed into a sulfate electrolyte consisting of H_2_SO_4_ and CuSO_4_. The anode was formed by an immersed copper block. When the copper had grown and micro-vias were filled, copper continued to grow and solderable copper pads protruding the surface of PI-Film formed (Figure 1a).

**Figure 4:**
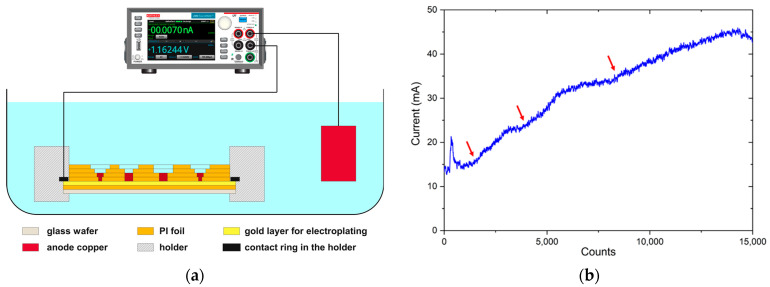
(**a**) Schematic illustration of the copper electroplating setup; (**b**) example of measured current over time at constant voltage of 600 mV. The counts in the diagram represent data points which were recorded with a frequency of approximately 12 measurements per second. The red arrows indicate the points where micro-via filling has just reached the next level of metallization.

The current source was operated in constant voltage mode for electroplating. Figure 4b shows a typical measurement of the current over the time of electroplating. The diagram represents a period of about 20 min. Three steps can be identified, which can be considered to represent the phase where the growing copper did not increase the contact area between metal and electrolyte in the micro-vias any further just before it reached the next level metallization, from what point on it increased again.

### 2.4. Laser Cutting

A femtosecond laser workstation (microSTRUCT C from 3D-Micromac, equipped with femtosecond laser Pharos from Light Conversion) emitting at 515 nm at 15 W (high voltage plus picker 90%, mark speed is 500 mm/s, pulse divide 1) was used to contour cut the finished sensor systems and to interrupt the electrical leads that were only required during electroplating (level 1). After laser ablation the sensors could be peeled off the glass wafer and became free to bend.

## 3. Results and Discussion

**Figure 5:**
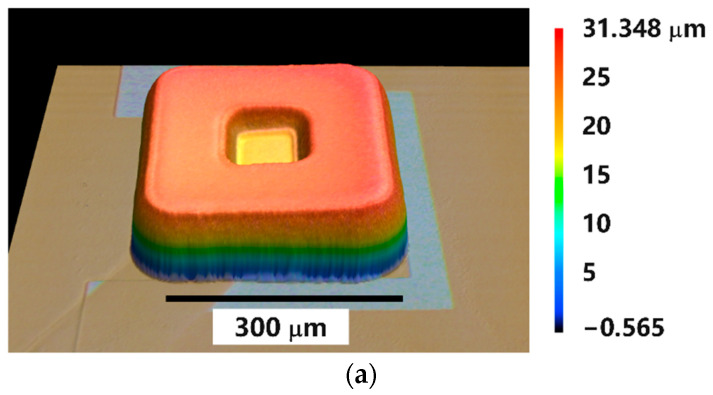

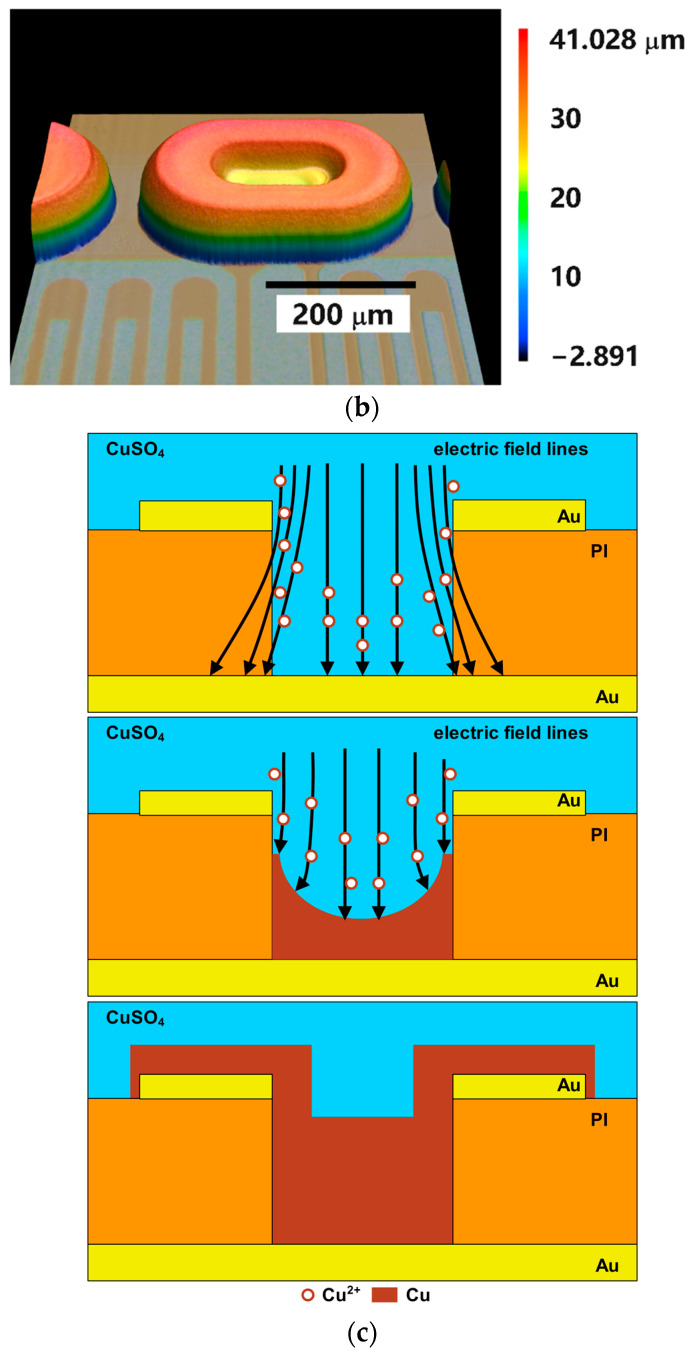
3D shape of the copper surface of contact pads (**a**) and filled micro-vias (**b**) as obtained by confocal laser scanning microscopy (VKX-260 from Keyence). (**c**) Schematic sketch illustrating the development of field lines and ion drifts during the electroplating of micro-vias and the result in shape of the copper filling.

The 3D images of the contact pads and of the micro-vias showed a smooth surface with a small central dent as exemplary shown in Figure 5. This dent results from the fact that the level 2 to level 4 metallizations were forming rings which attract the field lines and copper ions drifting more towards the walls of the hole during electroplating. This resulted in higher deposition rates at the edge than in the middle.

**Figure 6:**
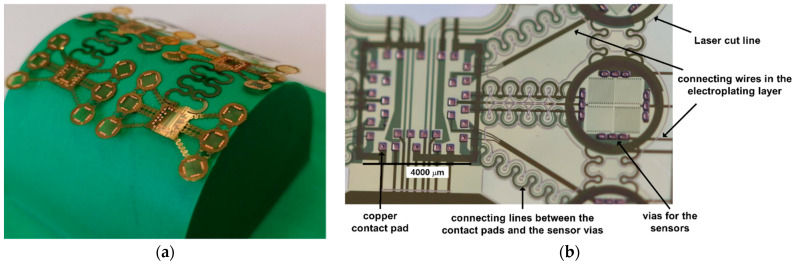
(**a**) Freely bendable sensor array was peeled off the glass wafer after laser cutting. (**b**) Detail view showing 32 contact pads in the area reserved for soldering the chip and 12 micro-vias in the area of one sensor node.

Once all the via-holes were covered with copper, the femtosecond laser was used to cut along the edge of the sensor. The sensor array can be carefully peeled off (Figure 6a). The straight metal lines in Figure 6b are from the level 1 metallization on the bottom polyimide layer that connect all the vias during electroplating. They were positioned in areas not occupied with other functional features and final separation with the laser could not disrupt the meandering wires on the other levels.

**Figure 7:**
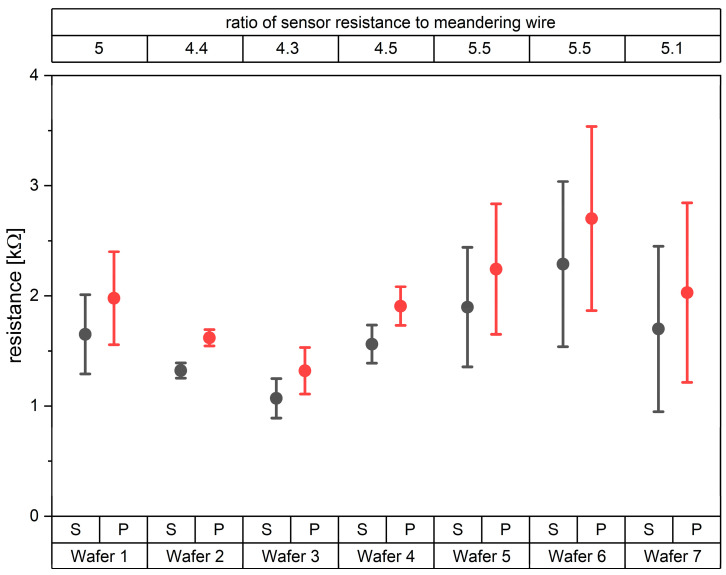
Averaged resistance values from seven wafers with standard deviations as measured at sensor vias (S) and pad (P). The variation in resistance for wafers 5–7 is high compared to wafers 1–4, which was caused by a less stable sputter process for these wafers.

On finished sensor foils still attached to the glass wafers the sensor resistances could be measured by contacting the four micro-vias at each sensor node. Contacting the contact pads for the chip integration, the sum of resistances of the sensors and of the wiring could be measured. In Figure 7 the measured resistance values obtained with seven fabricated wafers are shown. For each wafer 12 pairs of resistances were obtained. The differences between the measurements at vias and pads represented the resistances of the meandering wires that connect the sensor nodes with the chip. Even though the absolute values vary from wafer to wafer, the ratio of sensor resistance to meandering wire only varied between 4.3 and 5.5, which means the resistance recorded by the chip will be dominated by the strain sensor and not by the wires.

The sensors were bent in two directions while the voltage was measured between the copper contact surfaces shown in Figure 6b. A bend of +/− 120° was made, and the resulting voltage returned to the original value within 1.5% indicating that no damage was introduced.

## 4. Conclusions

We have successfully developed a novel four-level micro-via (4LµV) technology that produces solderable contact pads for flip chip soldering of ASICS and reliable interconnection in a flexible PI based temperature stable multilayer circuit. In comparison to known two-level-interconnections in flexible foils [2,7,10], our work represents a decisive improvement, because only in this way can complex and large-scale sensor arrays be realized with active electronics that allow multiplexing in the flexible foil. Our aim was to use PI film that can withstand temperatures of 360 °C without undergoing glass transition but must be cured at high temperatures during the fabrication. Compared to other substrate materials for flexible sensors like PEN or PET foils this is an important advantage. All other materials used are even more temperature stable. Our sensor arrays are used for respiration monitoring; the temperature is around 37 °C, and only slightly more when currents are applied. Therefore, we did not make tests at elevated temperatures, but as a perspective we want to highlight that applications in harsher environments may also be feasible with our technology. The filling of the micro-vias and establishing of the soldering pads by copper electroplating happens when all metallization layers have been established. This avoids any problems of copper oxidation during PI curing. A preliminary bending test showed already that in two bending directions the measured voltage turned to initial values, indicating that no damage occurred. As an example, for a multilevel system-in-foil a strain sensor array with 24 sensing positions was fabricated up to the point where the foil was ready for flip-chip solder mounting of a thin ASIC chip which turns the sensor array into an active system with multiplexed digital output, dramatically reducing external wiring complexity. Applications of this technology are not limited to this strain sensor array and the 4LµV technology can be used in future to create larger active arrays of a wide range of sensor types in formats of flexible foils with digital outputs.

## Data Availability

The raw data of the experiments can be requested from the authors.
